# Infection of ectocervical tissue and universal targeting of T-cells mediated by primary non-macrophage-tropic and highly macrophage-tropic HIV-1 R5 envelopes

**DOI:** 10.1186/s12977-015-0176-2

**Published:** 2015-06-09

**Authors:** Paul J Peters, Maria Paz Gonzalez-Perez, Thomas Musich, Tiffany A Moore Simas, Rongheng Lin, Abraham N Morse, Robin J Shattock, Cynthia A Derdeyn, Paul R Clapham

**Affiliations:** Program in Molecular Medicine and Department of Microbiology and Physiological Systems, University of Massachusetts Medical School, Biotech 2, 373 Plantation Street, Worcester, MA 01605-2377 USA; Department of Ob/Gyn, University of Massachusetts Memorial Health Care, 119 Belmont Street, Worcester, MA 01605 USA; School of Public Health and Health Sciences, University of Massachusetts, 411 Arnold House, 715 North Pleasant Street, Amherst, MA 01003-9304 USA; Department of Medicine, St Mary’s Campus, Imperial College, Medical School Building, London, W21PG UK; Department of Pathology and Laboratory Medicine, Emory Vaccine Center at Yerkes National Primate Center, Emory University, 954 Gatewood Road, Atlanta, GA 30329 USA

**Keywords:** Ectocervical explants, HIV-1 envelopes, Tropism, T-cells, Macrophages

## Abstract

**Background:**

HIV-1 variants carrying non-macrophage-tropic HIV-1 R5 envelopes (Envs) are predominantly transmitted and persist in immune tissue even in AIDS patients who have highly macrophage-tropic variants in the brain. Non-macrophage-tropic R5 Envs require high levels of CD4 for infection contrasting with macrophage-tropic Envs, which can efficiently mediate infection of cells via low CD4. Here, we investigated whether non-macrophage-tropic R5 Envs from the acute stage of infection (including transmitted/founder Env) mediated more efficient infection of ectocervical explant cultures compared to non-macrophage-tropic and highly macrophage-tropic R5 Envs from late disease.

**Results:**

We used Env+ pseudovirions that carried a GFP reporter gene to measure infection of the first cells targeted in ectocervical explant cultures. In straight titrations of Env+ pseudovirus supernatants, mac-tropic R5 Envs from late disease mediated slightly higher infectivities for ectocervical explants although this was not significant. Surprisingly, explant infection by several T/F/acute Envs was lower than for Envs from late disease. However, when infectivity for explants was corrected to account for differences in the overall infectivity of each Env+ pseudovirus (measured on highly permissive HeLa TZM-bl cells), non-mac-tropic early and late disease Env+ pseudoviruses mediated significantly higher infection. This observation suggests that cervical tissue preferentially supports non-mac-tropic Env+ viruses compared to mac-tropic viruses. Finally, we show that T-cells were the main targets for infection regardless of whether explants were stimulated with T-cell or monocyte/macrophage cytokines. There was no evidence of macrophage infection even for pseudovirions carrying highly mac-tropic Envs from brain tissue or for the highly mac-tropic, laboratory strain, BaL, which targeted T-cells in the explant tissue.

**Conclusions:**

Our data support ectocervical tissue as a favorable environment for non-mac-tropic HIV-1 R5 variants and emphasize the role of T-cells as initial targets for infection even for highly mac-tropic variants.

**Electronic supplementary material:**

The online version of this article (doi:10.1186/s12977-015-0176-2) contains supplementary material, which is available to authorized users.

## Background

HIV-1 R5 strains that use CCR5 as a coreceptor are predominantly transmitted [1] overcoming a tight bottleneck during heterosexual transmission. Frequently, only a single virus variant (often a minor variant in the donor) establishes infection in the new host [2]. HIV-1 R5 viruses infect both T-cells and macrophages and have been described as macrophage-tropic (M-tropic) [3]. However, many studies show that HIV-1 R5 viruses vary extensively in their ability to infect macrophages [4–12].

HIV-1 Env determinants previously identified to increase or modulate mac-tropism lie within or proximal to the CD4bs [13–15] and in the V1V2 and V3 loops [13, 16, 17]. Residues at these sites can enhance Env: CD4 interactions by several different mechanisms including (1) a direct increase in gp120:CD4 affinity [14, 15], (2) better access to the CD4 binding site (CD4bs) for CD4 [10] and (3) by enhancing the efficiency with which the Env trimer undergoes conformational changes induced by CD4 [18]. The ability of mac-tropic R5 Envs to efficiently interact with CD4 and trigger entry means that such Envs are more functional for entry compared to non-mac-tropic R5 Envs. The scarcity of mac-tropic R5 Envs in immune tissue and the periphery generally [5, 11, 19], strongly suggests that these highly functional Envs are selected against in vivo. It is possible that the increased exposure of the CD4bs on such Envs renders them sensitive to neutralizing antibodies, in immune tissue, although other mechanisms may also be involved [18].

The sequences of transmitted viruses have been deduced from multiple viral sequences that were PCR amplified from acute stage plasma of newly infected women, heterosexually and homosexually infected males and neonates [20–22]. Several studies of HIV-1 transmission events have investigated the properties of the envelope glycoproteins (Envs) of these transmitted/founder (T/F) viruses, while additional studies exploited HIV-1 *envs* derived from early, acute stage plasma by PCR [23–26]. These reports confirm that HIV-1 R5 viruses are predominantly transmitted and indicate that T/F Envs require high levels of CD4 for infection and do not confer efficient infection of macrophages [21, 22, 25, 27, 28]. Evidence from HIV-1 infection of ectocervical and other mucosal tissue explants [29–31] as well as SIV infection of macaques [32–34] demonstrate that initial cells targeted in mucosa are CD4+ T-cells consistent with transmission of non-mac-tropic R5 viruses.

Prior studies of cervical explant infections have shown no advantage for transmitted/founder/acute viruses over those from later in disease [31, 35]. However, these earlier studies were limited to small panels of viruses. In addition, they contained few primary R5 Envs that were highly mac-tropic and did not reveal whether preferential transmission of non-mac-tropic R5 variants is due to a cervical tissue block to infection by mac-tropic variants. It is therefore currently unclear whether mac-tropic R5 Envs can initiate infection of cervical tissue and whether they could preferentially target macrophages. The isolate, BaL has frequently been used as a prototype mac-tropic HIV-1 R5 isolate [31, 35]. However, this strain has been passaged through macrophages in vitro and is unlikely to be representative of primary mac-tropic envs derived directly from patient tissue.

Here, we compared a large panel (35 Envs) of HIV-1 T/F, acute and late stage non-mac-tropic R5 Envs with highly mac-tropic R5 Envs from late disease for infection of ectocervical explant cultures. The inclusion of a strong set of highly mac-tropic Envs thus allowed us to assess whether a transmission bottleneck for mac-tropic R5 HIV-1 acts at the level of cervical tissue infection and to assess whether such Envs confer infection of tissue macrophages in situ. We used Env+ pseudoviruses carrying GFP reporter genes to identify the initial cells targeted following infection of explants. In straight titrations of Env+ pseudoviruses, mac-tropic Envs mediated slightly higher infectivity for cervical explants, although this was not significant. However, when infectivity for explants was corrected to account for differences in the overall infectivity of Env+ pseudoviruses (measured on the highly permissive HeLa TZM-bl cells), non-mac-tropic early and late disease Env+ pseudoviruses mediated significantly more efficient infection. This observation suggests that cervical tissue preferentially supports non-mac-tropic Env+ viruses compared to mac-tropic viruses. Finally, we also confirm that T-cells are the universal initial target for infection in ectocervical tissue even for highly mac-tropic R5 Env+ viruses.

## Results

### T/F/acute Envs confer low levels of infection on primary macrophages

**Figure 1:**
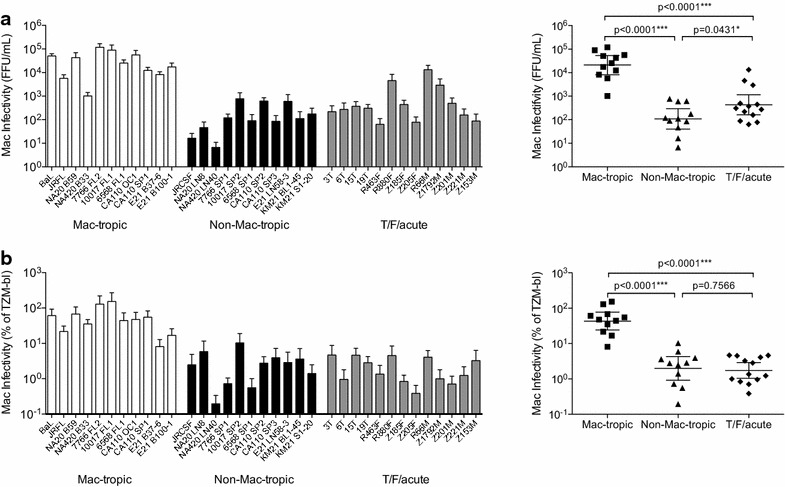
Macrophage infectivity for Envs selected for cervical explant studies. **a** Infectivity for macrophages (FFU/mL) for Env+ pseudoviruses (*left panel*). Mac-tropic Envs mediated significantly higher levels of macrophage infectivity compared to T/F/acute and late stage non-mac-tropic Envs (p values = <0.0001) *right panel*. **b** Macrophage infection plotted as a percent of TZM-bl infectivity (*left panel*). Correction of Env+ pseudovirus infectivity for different levels of infectivity on TZM-bl cells confirms that mac-tropic Envs mediated significantly higher levels of macrophage infectivity compared to T/F/acute and late stage non-mac-tropic Envs (p values = <0.0001) *right panel*. *Left panel* in **a** and **b** show means and standard errors from infectivities measured on macrophages derived from at least three donors, *right panels* show geometric means with 95% confidence intervals. p values were calculated using unpaired, two-tailed t tests in Prism 6.0f.

We first confirmed the levels of macrophage infectivity mediated by each of the different Envs to be studied here. Envs were derived from molecular clones of transmitted/founder (T/F) and acute stage *envs*, as well as late disease stage macrophage-tropic and non-macrophage-tropic envelopes (Table 1). We measured their capacity to confer infection of macrophages using GFP reporter pseudovirions (Figure 1). We first plotted infectivity as focus-forming units (FFUs) (GFP+ macrophages) to show the maximum infection for each Env+ pseudovirus (Figure 1a). However, to correct for different levels of Env+ pseudovirus infectivity (as revealed by titration on highly permissive TZM-bl cells), we also plotted infectivity for macrophages as a percent of TZM-bl infectivity (Figure 1b). As we reported previously [4, 5, 36, 37], pseudovirions carrying mac-tropic R5 Envs conferred significantly higher levels of macrophage infection (thousands of fold higher) compared to non-mac-tropic R5 Envs and most T/F and acute Envs. This is consistent with CD4+ T-cells (that express high amounts of CD4) being the major targets for infection by non-mac-tropic T/F viruses during transmission.

**Table 1:** HIV-1 *env* clones used to prepare GFP reporter Env+ pseudovirions

*env*	Clade	Full name	Origin	References
Transmitted/founder (T/F)				
3T	B	p1054.TC4.1499	Plasma	[20]
6T	B	p63358.p3.4013	Plasma	[20]
15T	B	p700010040.C9.4520	Plasma	[20]
19T	B	pPRB958_06.TB1.4305	Plasma	[20]
R463F	A1	R463FPL16MAR07EnvE44	Plasma	[49]
R880F	A1	R880FPL12JAN07EnvA6	Plasma	[49]
R66M	A/C	R66MPL7MAR06.3A9Env	Plasma	Unpublished
Z1792M	C	Z1792MPL18DEC07.3G7Env2	Plasma	Unpublished
Acute stage				
Z185F	C	Z185FPB24AUG02ENV3.1	Blood	[50, 51]
Z205F	C	Z205FPB27MAR03ENV1.1	Blood	[50–52]
Z201M	C	Z201MPL7FEB03ENV2.1	Plasma	[24]
Z221M	C	Z221MPL7MAR03ENV2.1	Plasma	[24, 50]
Z153M	C	Z153MPL13MAR02ENV5.1	Plasma	[24, 50]
Macrophage tropic				
BaL	B	BaL.26	Lung	[53]
JRFL	B		Brain	[6]
NA20 B59	B		Brain	[5]
NA420 B33	B		Brain	[5]
7766 FL2	B	7766 FL19-56-66	Brain	[19]
10017 FL1	B	10017 FL9-1-2	Brain	[19]
6568 FL1	B	6568 FL11-1-249	Brain	[19]
CA110 OC1	B	CA110 OC58-11-57	Brain	[19]
CA110 SP1	B	CA110 SP52-13-34	Spleen	[19]
E21 B37-6	B		Brain	[40]
E21 B100-1	B		Brain	[40]
Non-macrophage tropic				
JRCSF	B		CSF	[6]
NA20 LN8	B		Lymph Node	[5]
NA420 LN40	B	NA420 LN40/B33	Lymph Node	[5]
7766 SP1	B	7766 SP13-33-41	Spleen	[19]
10017 SP2	B	10017 SP10-9-65	Spleen	[19]
6568 SP1	B	6568 SP6-11-9	Spleen	[19]
CA110 SP2	B	CA110 SP53-23-131	Spleen	[19]
CA110 SP3	B	CA110 SP53-6-122	Spleen	[19]
E21 LN58-3	B		Lymph Node	[40]
KM21 BL1-45	G		Blood	[5]
KM21 S1-20	G		Semen	[5]

### Infection of ectocervical explant cultures using Env+ pseudovirions carrying a GFP reporter gene

**Figure 2:**
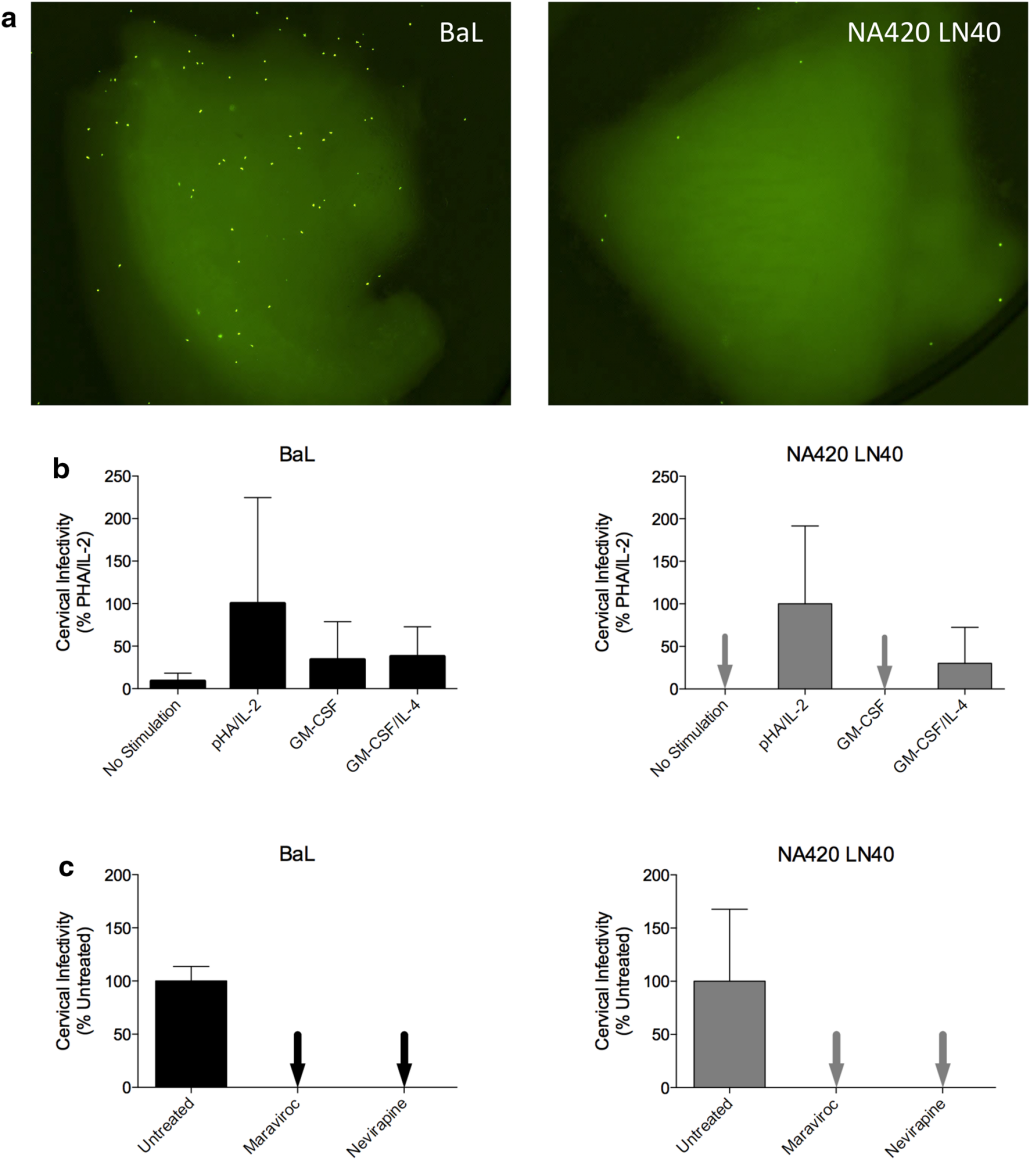
GFP reporter virus infection of ectocervical tissue explants. **a** Infected GFP+ cells in explant cultures. Infected, GFP+ cells are readily observed and easily distinguished from low-level auto-fluorescence of the tissue. Note some are GFP+ infected emigrant cells. **b** BaL and NA420 LN40 infection of explants without stimulation or in the presence of PHA/IL-2, GM-CSF, or GM-CSF and IL-4. Data are averaged from 2 or 3 replicate explants. **c** BaL and NA420 LN40 infection was efficiently inhibited following maraviroc blockade of CCR5 and by the post-entry, NNRTI inhibitor, nevirapine. Data are averaged from at least two donors with five replicate explants per donor.

We next evaluated the ability of Env+ pseudovirions to confer infection of primary ectocervical explant cultures (Figure 2). Infections were done on five replicates of each cervical tissue sample per donor and for at least five donors for each Env+ pseudovirion. There was a low level of autofluorescence in the explants and no fluorescence was present in the virus added. However, infected GFP+ cells became clearly visible after 4–5 days culture (Additional file 1: Figure S1) and were counted by microscopy on day 7 following infection (Figure 2a). Most infections were carried out in the presence of PHA and IL-2 stimulation, although stimulation with anti-CD3, anti-CD28 mabs resulted in similar levels of infection (not shown). In contrast, infection without stimulation resulted in infection on occasion, was mainly for BaL Env+ viruses and was at lower levels (Figure 2b). Infections in the presence of GM-CSF to support macrophage differentiation and activation also resulted in infection only on occasion. Similarly, infections in the presence of GM-CSF and IL-4 to support dendritic cell differentiation and maintenance also resulted in low or no infection. Infection with stimulation by GM-CSF or GM-CSF plus IL-4 was mainly with BaL+ viruses, and at similar levels to that observed without stimulation. Infection of PHA/IL-2 stimulated explants by BaL and non-mac-tropic R5 Env, LN40, was blocked by prior treatment with maraviroc (Figure 2c) verifying a CCR5-dependent route for infection. BaL and LN40 infectivity of cervical tissue were also blocked by the NNRTI nevirapine, a post-entry inhibitor (Figure 2c).

**Figure 3:**
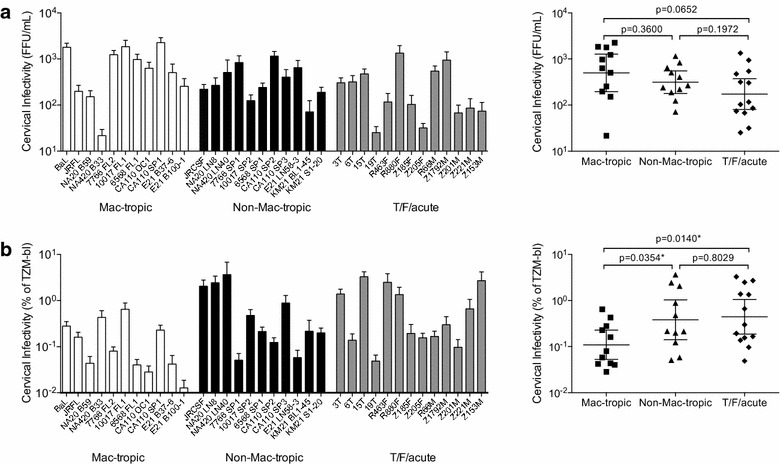
Ectocervical explant infectivity. **a** Ectocervical explant infection (FFU/mL) is shown for all Env+ pseudoviruses (*left panel*). No significant differences were detected between late disease mac-tropic, late-disease non-mac-tropic or T/F/acute Env+ pseudoviruses using unpaired, two-tailed t tests (*right panel*). **b** Infectivity for ectocervical explants plotted as a percent of HeLa TZM-bl infectivity. Using this correction, many T/F/acute and late disease non-mac-tropic Envs mediated higher infectivity (per TZM-bl IUs) compared to mac-tropic Envs (*left panel*). Ectocervical explant infectivity for late-disease non-mac-tropic and T/F/acute Env+ pseudoviruses was significantly higher than late-disease mac-tropic Envs (*right panel*). Each Env+ pseudovirus was tested on explants prepared from at least five donors with five replicate explants per donor.

We evaluated the maximum infectivity mediated by each Env by directly titrating each Env+ pseudovirion preparation on explants and estimating their infectivity titers by counting infected GFP+ cells. Env+ pseudovirions (Table 1) were titrated on ectocervical tissue explants in groups of up to 23 in parallel with standards, JR-FL, JR-CSF and BaL Env+ pseudovirions. The highly mac-tropic BaL Env consistently mediated the highest infectivity for PHA/IL-2 stimulated explants. We found variable levels of infection of ectocervical explants within all three groups of Envs, T/F/acute, late disease stage mac-tropic and late disease non-mac-tropic (Figure 3a). Overall, there was no statistical difference in the ectocervical explant infection between mac-tropic and non-mac-tropic R5 Envs from late infection, or T/F/acute Envs. However, surprisingly, the geometric mean of infection for T/F/acute Envs was the lowest.

In a second analysis, we normalized ectocervical explant infectivity titers to their titers measured on the highly permissive HeLa TZM-bl cell line to correct for variation in overall Env+ pseudovirus infectivity. All Env+ pseudoviruses had respectable titers on TZM-bl cells, although some variation was noted. For example, we previously reported that mac-tropic R5 Envs generally mediated higher levels of infectivity for all CD4+ cells compared to non-mac-tropic Envs [37], including HeLa TZM-bl cells, although this was much more marginal compared to primary macrophages. As discussed in the Background section, the ability of mac-tropic Envs to bind CD4 efficiently and use low levels of CD4 for infection indicates that these Envs are probably more functional during entry compared to non-mac-tropic Envs.

When we plotted ectocervical infectivity titers corrected for TZM-bl infectivity, we found that non-mac-tropic Envs (both T/F/acute and late disease) mediated significantly higher explant infection than mac-tropic Envs (Figure 3b). This analysis suggests that cervical tissue preferentially supports infection mediated by non-mac-tropic Envs. This observation is intriguing. However, it should be emphasized that non-mac-tropic R5 Envs did not confer higher levels of infection of explants overall (Figure 3a). Still, these observations suggest that cervical tissue provides an environment that is favorable for HIV-1 carrying non-mac-tropic Envs (including T/F Envs) during transmission.

### Identification of the infected GFP+ cells

**Figure 4:**
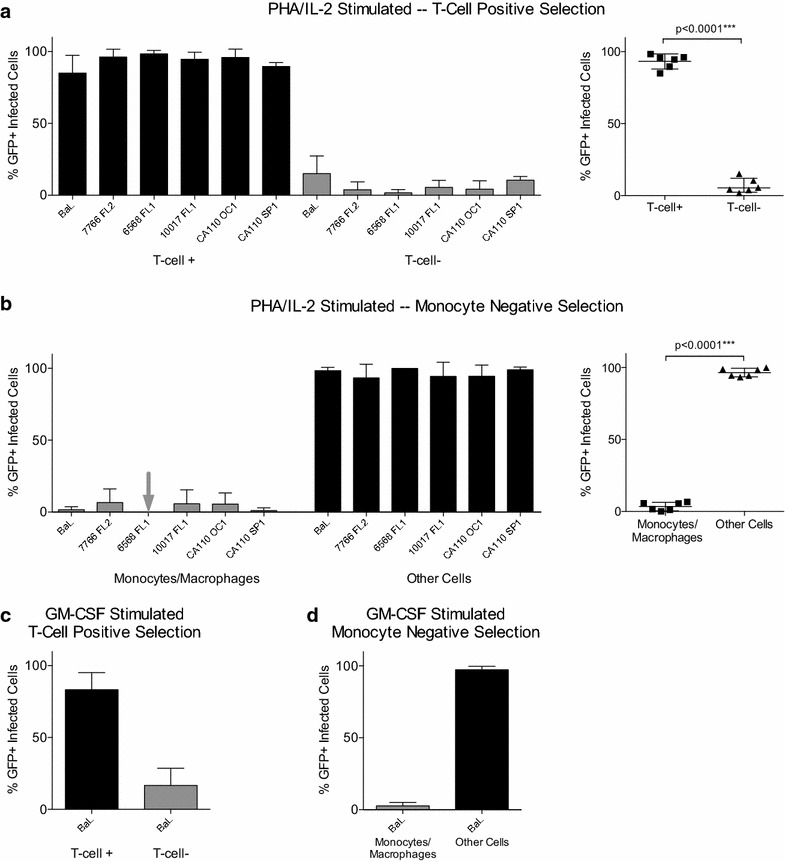
T-cells are the major cell type infected in ectocervical explants. Cells extracted from PHA/IL-2 stimulated, infected explant cultures were subjected to positive selection of T-cells (**a**) or negative selection for monocytes and macrophages (**b**) using StemCell Technologies EasySep immunomagnetic kits. Infected GFP+ cells consistently segregated with T-cells and not monocyte/macrophages. In other experiments, BaL infected explants were stimulated with GM-CSF and cultured in human AB+ plasma to support monocyte/macrophages. Extracted cells then underwent T-cell positive selection (**c**) or monocyte/macrophage negative selection (**d**). Again, GFP+ cells segregated with T-cells and not monocyte/macrophages.

We used an immunomagnetic selection to separate GFP+ infected cells from explant cultures into T-cell or monocyte/macrophage fractions as described in “Methods”. We included the cells present in collagenase digested explant cell suspensions as well as emigrant cells that had migrated out of the explant. We focused on infections using pseudoviruses that carried highly mac-tropic Envs to provide the best chance of detecting infection of cells other than T-cells. Using this approach, we clearly demonstrated that T-cells were the main cell type infected in PHA/IL-2 stimulated ectocervical explant cultures (Figure 4). Positive selection of T-cells pulled out the vast majority of GFP+ cells from explants infected with lab-adapted mac-tropic BaL or primary mac-tropic R5 Envs 7766 FL2, 6568 FL1, 10017 FL1, CA110 OC1 and CA110 SP1 (Figure 4). A minority of GFP+ cells was detected in the unbound fraction and it is unclear what these cells are. However, since very few GFP+ cells were detected in monocyte/macrophage-enriched fractions (Figure 4b), they are unlikely to be macrophages and are most likely infected T-cells that have leaked through the positive selection strategy. In some experiments, we investigated only the GFP+ emigrant cells from PHA/IL-2 stimulated explants and these cells were also predominantly T-cells (Additional file 2: Figure S2).

For most experiments, explants were cultured in the presence of PHA and IL-2, which is likely to greatly increase the numbers of target T-cells as well as enhance their susceptibility. However, we also infected explants that were stimulated with GM-CSF and cultured in the presence of human plasma to support macrophage differentiation and activation. In these experiments, infectivity was low with only BaL Env+ pseudoviruses mediating significant infection. Immunomagnetic separation of explant cells showed that T-cells were the predominant cell type infected in these cultures (Figure 4c, d).

## Discussion

Here, we investigated the capacity of HIV-1 R5 Envs to confer infection of ectocervical explant tissue. We used Env+ pseudoviruses to specifically focus on the first cells infected. We show that a range of HIV-1 Envs designated as T/F/acute or late stage non-mac-tropic and mac-tropic R5 Envs all mediated infection, although the extent of infection varied. Although ectocervical mucosa have been shown to contain abundant macrophages, dendritic cells and T-cells [38], we confirm that T-cells are the major target for HIV-1 infection in cervical tissue [30, 31], even for mac-tropic Envs.

We took two approaches to assess the infectivity of mac-tropic and non-mac-tropic R5 Env+ pseudoviruses. First, we titrated different Env+ pseudoviruses on explants to compare the maximum infectivity mediated by each Env. This approach showed that mac-tropic Envs mediated the highest average infectivity although this was not significant (Figure 3a).

In a second approach, we standardized our ectocervical infectivity data by plotting them as percentages of their titers on the highly permissive HeLa TZM-bl cell line as we previously reported [4, 5, 37]. This latter approach corrects for variation in the amount of infectious virus produced by 293T cells. For example, while all Env+ pseudoviruses mediated respectable infectivity titers on HeLa TZM-bl cells, some of the non-mac-tropic Envs mediated lower levels of infectivity for these cells [5, 37]. Plotting ectocervical infectivity as a percent of TZM-bl infectivity showed that many non-mac-tropic Envs were more efficient for explant infection compared to mac-tropic Envs i.e. they required less input of virus infectivity (measured on TZM-bl cells) to mediate the same level of explant infection as mac-tropic Envs (Figure 3b). Although this observation is interesting, it does not override the higher maximal infectivities mediated by mac-tropic Env+ viruses (Figure 3a) since Envs that confer less infectious virions in vitro are also likely to produce less infectious virions in vivo. Nevertheless, this result does suggest that cervical tissue provides preferential support for non-mac-tropic Env+ viruses.

We show that cervical tissue explants are relatively insensitive to infection and usually require stimulation before infection can be detected. This is not surprising when considering the inefficiency of male-to-female HIV transmission [39]. Infection of cervical tissue in the absence of stimulation was only observed occasionally. We suspect that T-cells in these (permissive) tissues may already be activated, but did not measure that. We compared other stimulation protocols to activate macrophages and DCs, in addition to T-cells. However, only T-cell stimulation resulted in consistent infection. Our data supports the idea that T-cell stimulation (e.g. caused by inflammation resulting from other STD infections) in the female genital tract, would significantly increase the likelihood of HIV transmission. However, they also suggest that such inflammation may not increase the susceptibility of other potential target cells including macrophages and DCs.

Our study is the first that has investigated a large number of primary R5 Envs for infection of cervical tissue including a number that are highly mac-tropic. Highly mac-tropic R5 Envs have been detected in semen and will thus have the opportunity for mucosal transmission [36, 40]. In addition, the NA20 B59 Env and other primary R5 Envs have previously been shown to mediate infection and replication in macrophages derived from cervical tissue [41], indicating such cells are permissive. All the highly mac-tropic Envs conferred infection of cervical explants. However, infection was restricted to T-cells with no evidence of macrophage infection by the highly mac-tropic BaL or by primary R5 Envs, even when conditions favored macrophages. The lack of observed macrophage infection could be due to the low overall sensitivity of the cervical tissue infectivity system. However, we cannot rule out the presence of a putative intrinsic block for infection of cervical macrophages in situ. For example, an Env dependent post-entry restriction in cervical macrophages has been described [42]. Also, innate immune activation of cervical macrophages may prevent HIV infection [43]. This could allow entry of HIV pseudovirions into cervical macrophages but prevent production of the LTR-dependent GFP reporter. Regardless, our data show emphatically that there is no selective block to highly mac-tropic R5 viruses carrying primary Envs for infection of cervical tissue.

Our study focused on cell-free virus infection of ectocervical tissue and did not address the possibility that HIV-1-infected cells present in semen may mediate infection of target cells in the vagina or cervix. Nor did we investigate whether macrophages became infected following the spread of infectivity by replication competent viruses. Both of these are possibilities and require further study.

Our study did not reveal the roles of myeloid DCs or trans-infection in explant infection. When purified PHA/IL-2 stimulated CD4+ T-cells are infected with cell free GFP reporter viruses in vitro, infected GFP+ cells are fully visible within 2 days after infection. Here, GFP+ T-cells were only apparent in explants after 4–5 days following infection. It is tempting to speculate that the delay involves sequestration by DCs and their interactions with T-cells. In support of this, Shen et al. [44] reported that DCs in vaginal and ectocervical mononuclear cells were the first to take up GFP-tagged virions. However, we have not yet been able to confirm a role for DCs in our assays.

## Conclusions

In summary, our data show that R5 Envs that vary extensively in tropism mediate similar levels of ectocervical explant infection and universally target T-cells. There is no selective barrier for highly mac-tropic R5 viruses even though T-cells rather than macrophages are targeted. However, our data suggest that cervical tissue preferentially supports infection mediated by non-mac-tropic R5 Envs generally, including T/F and acute stage R5 Envs as well as those from late disease.

## Methods

### Viruses

We selected a panel of HIV-1 R5 *envs* that were derived from transmitted/founder (T/F) and acute stage viruses and included macrophage-tropic and non-macrophage-tropic R5 *envs* from AIDS patients in late disease (Table 1). *Env* or *rev*-*env* sequences were cloned into pSVIIIenv or pcDNA™ 3.1D/V5-His-TOPO® (Invitrogen Inc.) respectively. Pseudoviruses were prepared by cotransfection of 293T cells with an *Env+* vector, *env*-minus pNL4.3 and a GFP reporter vector, pHIVec2/GFP [45] at a ratio of 1:1:1.5. The cell supernatant was changed 8–18 h post-transfection (4% FBS DMEM). Pseudovirions were harvested 48 h post-transfection, clarified by low-speed centrifugation, aliquoted into 0.5–1.0-mL portions, and snap-frozen in liquid nitrogen.

### Cells

293T cells were used to prepare Env-containing (Env+) pseudovirions by transfection. Env+ pseudovirions were initially titrated on HeLa TZM-bl cells [46]. 293T and HeLa TZM-bl cells were cultured in Dulbecco modified Eagle medium (DMEM) with 4% fetal bovine serum (FBS) and gentamicin (10 μg/mL).

Primary macrophages were prepared from blood monocytes. Briefly, 3 × 10^7^ elutriated monocytes were plated into 15 cm bacterial petri dishes and cultured in 10% human AB+ plasma in DMEM for 5–7 days. Alternatively, 5 × 10^7^ Ficoll-purified peripheral blood mononuclear cells (PBMC) from a buffy coat (Research Blood Components LLC, Boston, MA) were plated into 15-cm bacterial culture dishes for 3 h before extensively washing away non-adherent cells, culturing overnight, and repeating the washes. The adhered monocytes were then cultured for 5–7 days in 10% human plasma in DMEM. The differentiated macrophages were treated with EDTA and transferred to 48-well tissue culture dishes the day prior to infection at 1.25 × 10^5^ cell/well [4, 5].

### Infectivity assays

We measured the infectivity of Env+ pseudoviruses for HeLa TZM-bl, macrophages and cervical explant cultures (see below for details). We chose HeLa TZM-bl cells over PBMCs or primary T-cells to standardize Env+ pseudovirus infectivity. Our study focuses on mac-tropic and non-mac-tropic R5 Envs, which may vary in their ability to infect CD4+ T-cells, the main targets for infection in PBMCs. HeLa TZM-bl cells express high levels of CD4 and CCR5 and are highly permissive to a wide range of HIV-1 variants and isolates. They are stable and provide standard and repeatable reference data for infectivity measurements.

We titrated viruses in 10-fold dilutions on each target cell type. We titrated pseudoviruses on TZM-bl cells (without DEAE Dextran). We used the TZM-bl titers to standardize the infectivity of Env+ pseudoviruses on macrophages and cervical explant cultures. Here, we have presented primary infectivity data for macrophages and cervical tissue along with infectivity titers plotted as a percentage of the infectivity recorded on HeLa TZM-bl cells. The former approach reveals the maximum infectivity possible for each Env, while the latter protocol corrects for Env+ pseudovirus differences in HeLa TZM-bl infectivity. We have used this approach previously [5]. It avoids having to equilibrate viruses to the same infectivity titers, a process that can introduce additional error and limits the maximum infectivity used, to that of the virus with the lowest infectivity. It therefore allows us to cover the maximum range of Env+ pseudovirus infectivity of cervical tissue explants.

### Macrophage infectivity

Macrophage infectivity was assessed on duplicate wells of at least 2 batches of macrophages from independent donors. Macrophages seeded in 48 well plates were pretreated with 100 µL DEAE dextran (10 µg/mL) in DMEM medium containing 10% human plasma for 30 min at 37°C before Env+ pseudoviruses carrying a GFP reporter gene were added at 100 µL/well. Infected plates were spinoculated for 45 min at 1,200 RPM in a benchtop centrifuge at room temperature [47]. Infected macrophages were incubated for a further 3 h at 37°C before the addition of 300 µL of DMEM (10% human plasma) and incubating at 37°C for 7 days. DEAE dextran and spinoculation enhance virus infectivity by approximately 20-fold by increasing attachment [47] and entry [48]. Infection following this procedure does not bypass the requirement of CD4 and CCR5 for infection, which remains sensitive to entry inhibitors including maraviroc (not shown). Env^+^ pseudovirions are capable of only a single round of replication so that focus-forming units (FFU) were estimated 5–7 days post-infection by counting infected GFP+ macrophages by fluorescent microscopy.

### Ectocervical explant infectivity

Ectocervical explants were prepared from fresh tissue provided without identifiers from hysterectomy surgeries performed earlier in the day. Tissue came from subjects whose prior Papanicolaou test (Pap smear) was normal. Small 2 mm^3^ pieces were seeded into 96 well plates, washed in growth medium [RPMI 1,640, 10% heat inactivated fetal calf serum with l-glutamine (2 mM), penicillin (50U/mL) and streptomycin (50 μg/mL)] and infected immediately. 100 μL of Env+ pseudoviruses was added to explants in growth medium containing 5 μg/mL phytohemagglutinin and after 2 days, IL-2 (5 ng/mL, Roche Inc.). In some experiments, explants were infected without stimulation, with stimulation using anti-CD3/antiCD28 Dynabeads (Life Technologies Inc.), in the presence of GM-CSF (100 ng/mL) to support monocytes and macrophages, or in the presence of GM-CSF (100 ng/mL) and IL-4 (40 ng/mL) to support dendritic cells. Stimulation with the anti-CD3/antiCD28 Dynabeads gave similar results as stimulation with PHA/IL-2 (not shown). Infections were done on five replicate wells for at least five donors. GFP+ cells were quantified by fluorescent microscopy after 7 days, by counting all the GFP+ cells in a well including those within the explant and the emigrants that had migrated from the tissue and were present on the bottom of wells. Inhibition assays were done with 100 ng/mL maraviroc or 2 μM nevirapine added prior to infection and maintained until infectivity was read.

### Evaluation of the cell types of GFP+ cells in explants

GFP+ cells were present within the explant, although many had emigrated into the culture well and surrounded the piece of cervical tissue following 7 days culture. To evaluate whether the GFP+ cells were T-cells or macrophages, we digested explants using collagenase Type IV (Gibco, Life Technologies) and pooled released cells with emigrants. In some assays we collected only the emigrant cells. We then used StemCell EasySep immunomagnetic positive selection and enrichment protocols. The Human CD3 Positive Selection Kit (typically achieves 99.4–99.8% purity from fresh peripheral blood mononuclear cells) was used to select for T-cells. While the Human Monocyte Enrichment Kit (negative selection, typically achieves 83–95% purity from previously frozen peripheral blood mononuclear cells), this protocol was chosen for monocytes/macrophages. This is because ectocervical tissue macrophages were reported to not express CD14 [41], which is the target for positive selection kits. Selected and depleted cell populations were evaluated for GFP+ cells by fluorescent microscopy.

### Statistical analyses

Macrophage infectivity and ectocervical explant infectivity were evaluated in an unmodified format (FFU/mL) and a normalized format (% of TZM-bl). In both cases the data were summarized as mean and standard error of the mean for each pseudovirus. Comparisons were also made among three groups of pseudoviruses, late-disease macrophage-tropic, late-disease non-macrophage-tropic, and transmitted/founder/acute. To achieve normality within groups the data were log transformed. Then groups were compared by unpaired two-tailed t test in Prism 6.0f. Groups are presented with their geometric mean and 95% confidence intervals (Figures 1, 3).

Data for ectocervical explant stimulation methods and maraviroc and nevirapine treatment are mean and standard error (Figure 2). Data for ectocervical cell selections are presented with mean and 95% confidence intervals with group comparisons tested by the Wilcoxon matched-pairs signed rank test in Prism 6.0f (Figure 4).

## Additional files

Additional file 1:
**Figure S1.** GFP+ infected cells in ectocervical explants become visible by day 6. GFP+ infected cells in a BaL infected ectocervical explant are clearly visible at day 6, but not at day 2 or 3. Additional File 2:
**Figure S2.** T-cells are the major cell type infected among cells that have emigrated from ectocervical explants. Emigrant cells from PHA/IL-2 stimulated, infected explant cultures were subjected to positive selection of T-cells (A) or negative selection for monocytes and macrophages (B) using StemCell Technologies EasySep immunomagnetic kits.
