# Supplementary figures and images for: Infection of ectocervical tissue and universal targeting of T-cells mediated by primary non-macrophage-tropic and highly macrophage-tropic HIV-1 R5 envelopes
Source: Retrovirology. 2015 Jun 9;12:48. doi: 10.1186/s12977-015-0176-2 (PMC4459458; doi:10.1186/s12977-015-0176-2)

Figure S1

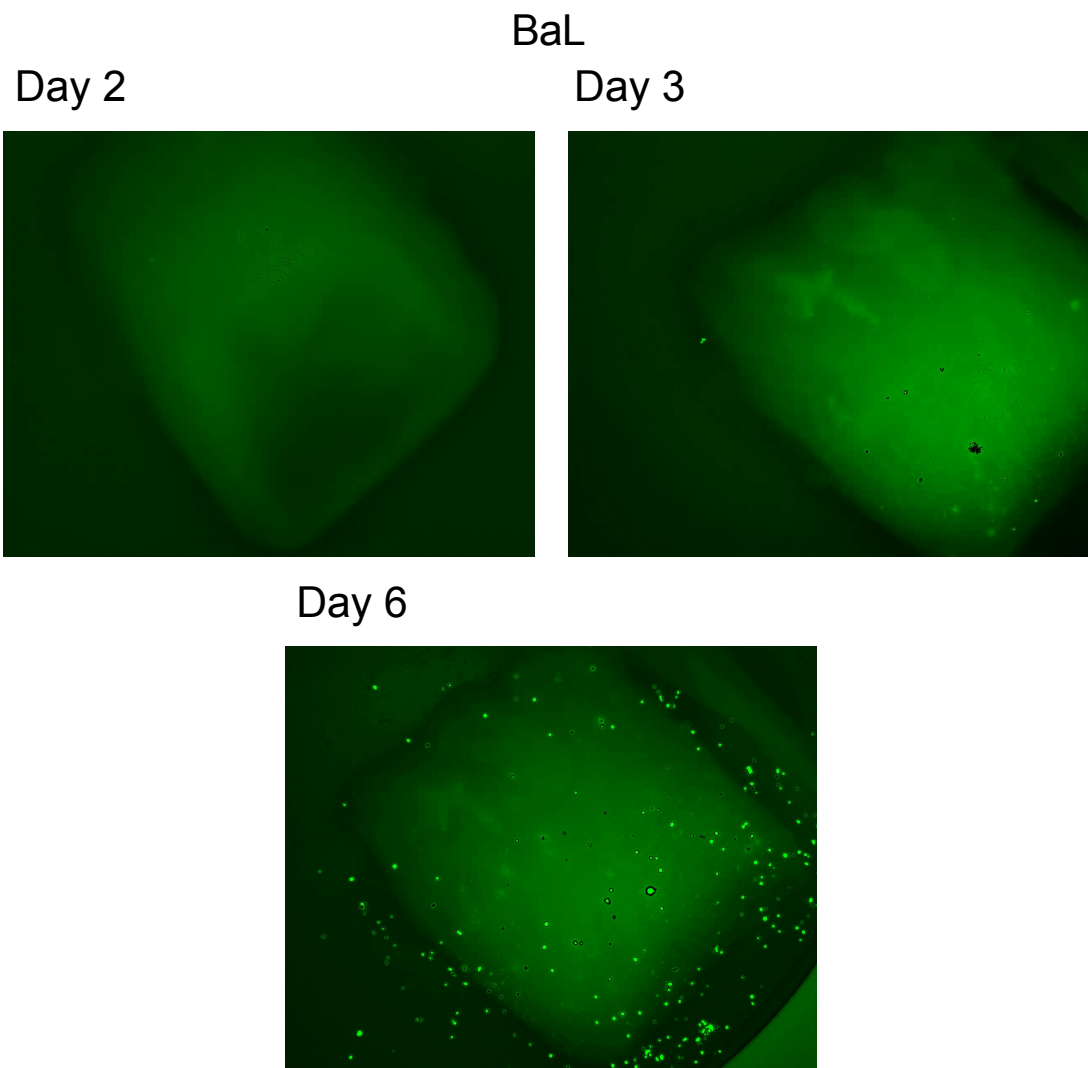

Supplement: Additional file 1: — Figure S1. GFP+ infected cells in ectocervical explants become visible by day 6. GFP+ infected cells in a BaL infected ectocervical explant are clearly visible at day 6, but not at day 2 or 3. [file 12977_2015_176_MOESM1_ESM.pdf]
