# Supplementary material for: Infection of ectocervical tissue and universal targeting of T-cells mediated by primary non-macrophage-tropic and highly macrophage-tropic HIV-1 R5 envelopes
Source: Retrovirology. 2015 Jun 9;12:48. doi: 10.1186/s12977-015-0176-2 (PMC4459458; doi:10.1186/s12977-015-0176-2)

Figure S2

A Emigrant Cells: PHA/IL-2 Stimulated -- T-Cell Positive Selection

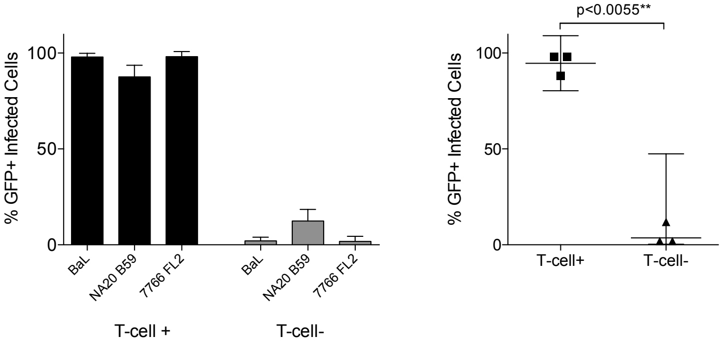

B Emigrant Cells: PHA/IL-2 Stimulated -- Monocyte Negative Selection

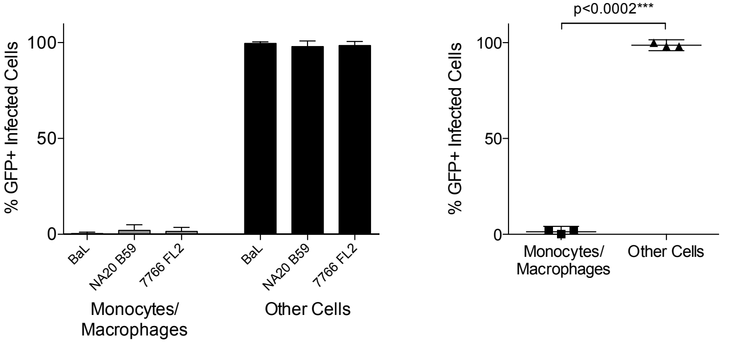

Supplement: Additional File 2: — Figure S2. T-cells are the major cell type infected among cells that have emigrated from ectocervical explants. Emigrant cells from PHA/IL-2 stimulated, infected explant cultures were subjected to positive selection of T-cells (A) or negative selection for monocytes and macrophages (B) using StemCell Technologies EasySep immunomagnetic kits. [file 12977_2015_176_MOESM2_ESM.pdf]
